# Shallow vestibular depth as a risk indicator for peri-implantitis: a retrospective analysis of 336 implants

**DOI:** 10.1007/s00784-026-06807-w

**Published:** 2026-03-19

**Authors:** Bülent Ulaştan, Ahmet Dağ, Hatice Ortaç

**Affiliations:** 1 Department of Periodontology, Faculty of Dentistry, Dicle University, Diyarbakır, Türkiye; 2 Department of Biostatistics, Faculty of Medicine, Dicle University, Diyarbakır, Türkiye

**Keywords:** Vestibule depth, Peri-implant tissues, Peri-implantitis, Peri-implant phenotype

## Abstract

**Objective:**

This retrospective cross-sectional study aimed to evaluate the relationship between vestibular depth (VD) and peri-implant soft tissue parameters, marginal bone loss (MBL), and peri-implant disease status.

**Materials and methods:**

A total of 336 implants from 65 patients (27 males, 38 females) who had at least one implant restored prosthetically and whose implants had been in function for at least 1 year were examined at the Department of Periodontology, Faculty of Dentistry, Dicle University. Demographic and clinical data (age, gender, smoking, oral hygiene, implant function duration) were recorded. In the clinical evaluation, VD, keratinized mucosa (KM) width and phenotype, and periodontal/peri-implant parameters (modified plaque index (mPI), modified gingival index (mGI), probing pocket depth (PD), bleeding on probing (BOP), gingival recession, and suppuration) were recorded. Implants were divided into two groups based on VD: shallow VD (≤ 4 mm) and adequate VD (> 4 mm). The primary endpoint was the presence (yes/no) of peri-implantitis at the implant level according to clinical and radiographic criteria, reported as prevalence in the study population. Radiographic bone loss was measured on panoramic radiographs. The VD groups were compared using the Mann–Whitney U and χ² tests; within-patient clustering was accounted for using GEE logistic regression.

**Findings:**

A total of 336 implants from 65 patients were evaluated. VD ≤ 4 mm was detected in 26.5% of implants, and > 4 mm in 73.5%. According to clinical diagnosis, 39.9% of implants (*n* = 134) had peri-implant health, 34.5% (*n* = 116) had peri-implant mucositis, and 25.6% (*n* = 86) had peri-implantitis. In the shallow VD group, mPI, mGI, BOP, PD, gingival recession, radiographic bone loss, and suppuration were found to be higher than in the adequate VD group in exploratory bivariate analyses (all *p* < 0.05). In the multivariate GEE analysis, VD ≤ 4 mm was independently associated with peri-implantitis (OR = 2.405; 95% CI: 1.233–4.690; *p* = 0.010). Additionally, the odds of peri-implantitis were increased in those with implant duration of 5–10 years (OR = 4.026; *p* = 0.044), and the maxillary anterior region showed a lower likelihood of peri-implantitis compared to the mandibular posterior region (OR = 0.409; *p* = 0.026).

**Conclusion:**

Shallow VD was associated with gingival recession, MBL, and the presence of peri-implantitis, along with higher plaque accumulation and soft tissue inflammation. The findings suggest that adequate VD, as a component of the peri-implant phenotype, may be associated with the maintenance of peri-implant health. In particular, in shallow vestibules with a KM width < 2 mm and a thin mucosal phenotype, approaches such as vestibuloplasty and/or soft tissue augmentation may be considered in selected cases to support plaque control.

**Clinical significance:**

Our findings suggest that evaluating VD as part of the peri-implant phenotype may help identify conditions that make maintaining hygiene difficult in areas with shallow vestibules and facilitate discussion of mucogingival approach options in selected cases.

## Introduction

 Dental implants are one of the most commonly preferred treatment options today for the rehabilitation of missing teeth. Advances in surgical techniques and improvements in implant surface properties have resulted in high success rates. Thanks to these positive outcomes and long-term survival rates, dental implants have become one of the most widely accepted treatments among both patients and clinicians [1]. However, clinicians are paying increasing attention to epidemiological studies reporting peri-implant inflammatory diseases, particularly peri-implantitis [2].

The 2017 classification of peri-implant diseases, jointly published by the American Academy of Periodontology and the European Federation of Periodontology, defines peri-implant health, peri-implant mucositis, peri-implantitis, and peri-implant soft and hard tissue defects. According to this consensus, peri-implant health is characterized by the absence of peri-implant mucositis and peri-implantitis, and clinically, erythema, swelling, or suppuration are not expected. According to the current report by Tonetti et al. [3] although isolated bleeding at a single site that is not profuse is considered acceptable in a clinically healthy state, the absence of BOP is preferred. Peri-implant mucositis is defined by the presence of clinically evident inflammation, while peri-implantitis is characterized by inflammation of the peri-implant mucosa followed by progressive loss of supporting bone; typically presenting with increased PD (PD ≥ 6 mm) accompanied by bleeding and/or suppuration on probing and radiographic evidence of bone loss [4, 5].

Peri-implant diseases are a common global problem in implant patients and pose a significant challenge in modern implant dentistry; the main reasons for this are the need for early diagnosis/regular follow-up and the variable/unpredictable outcomes of peri-implantitis treatment [6]. In a recent systematic review and meta-analysis based on the 2017 World Workshop criteria, the weighted mean prevalence values calculated by meta-analysis were reported as 63.0% at the patient level and 59.2% at the implant level for peri-implant mucositis; and 25.0% at the patient level and 18.0% at the implant level for peri-implantitis [7].

The long-term success and sustainability of dental implants are closely related to the health and stability of peri-implant tissues. Matarasso et al. [8], reported approximately 96% survival in implants at 10-year follow-up, emphasizing the importance of MBL stability in maintaining long-term clinical success. Similarly, Iorio-Siciliano et al. [9], drew attention to the relationship between peri-implant soft tissue conditions and MBL stability in long-term follow-up.

According to the 2017 World Congress of Periodontology [10], the main risk factors for peri-implant diseases include a history of periodontitis, inadequate plaque control, and inadequate maintenance treatment. Various predisposing factors associated with peri-implantitis and facilitating biofilm accumulation have been identified; one of these is VD [11].

VD is defined as the distance between the coronal margin of the attached gingiva and the deepest point of the mucobuccal fold [12]. Adequate VD is generally considered necessary for maintaining proper oral hygiene [13]. It is thought that the combination of a narrow keratinized mucosal band and shallow VD facilitates food impaction during mastication and makes effective oral hygiene difficult [14]. Therefore, vestibuloplasty/mucogingival procedures aimed at increasing the VD may be considered to improve patient comfort and facilitate plaque control, particularly in selected cases where discomfort is reported during brushing and/or hygiene cannot be maintained [15].

The 2017 periodontal disease classification suggested that a shallower vestibular fornix may be associated with soft tissue inflammation, which can make plaque removal difficult and thus hinder effective oral hygiene [16]. Furthermore, sufficient VD in areas adjacent to dental implants has been reported as a parameter that may be related to long-term implant outcomes [17].

The number of studies in the literature that jointly address the relationship between VD peri-implant soft tissue parameters, MBL, and peri-implant disease status appears to be limited. The current literature focuses more on KM width and peri-implant phenotype, with VD mostly reported as a secondary or observational finding. This study aims to evaluate the possible relationship between VD and peri-implant soft tissue health, radiographic bone loss, and peri-implant diseases. It is thought that the findings will contribute to the evidence-based definition of this clinically important morphological parameter.

## Materials and methods

###  Ethical approval

 This study was conducted in accordance with the principles of the Declaration of Helsinki. The study protocol was approved by the Ethics Committee of Dicle University Faculty of Dentistry (Approval no: 2024-24, date: September 25, 2024). There is no clinical trial number.

###  Patient sample

Following ethical committee approval, consecutive patients who applied to the Department of Periodontology, Faculty of Dentistry, Dicle University between January 6, 2025, and May 29, 2025, and who met the inclusion criteria were included in this retrospective cross-sectional study. Inclusion criteria were: being ≥ 18 years of age and having at least one implant restored with a prosthetic restoration that had been in function for at least one year. Exclusion criteria were: being younger than 18 years of age, having undergone periodontal treatment within the last six months, implants with unreliable assessment of reference points and crestal bone level due to blurring/motion artifacts or superposition–distortion, implants for which implant length information could not be obtained (calibration could not be performed), refusal to participate in the study, pregnancy, ongoing radiotherapy or chemotherapy, systemic antibiotic use within the last three months, use of immunosuppressive drugs, and the presence of prosthetic restorations that physically restrict probe placement in a way that would interfere with PD/BOP measurement (to reduce measurement error and potential misclassification).

A total of 418 implants were evaluated; however, 336 implants with complete clinical and radiographic data were included in the analysis. Implants with missing records or radiographs unsuitable for measurement were excluded from the analysis; imputation was not applied, and analyses were performed using the complete-case approach.

The sample size was determined based on a preliminary power analysis conducted using the differences in peri-implant parameters reported in the reference study [13]. Assuming a two-tailed Type I error level of 5% (α = 0.05) and a planned test power of 95% (1–β = 0.95), it was calculated that including at least 290 implants in the study would be sufficient. The 336 implants evaluated in this study exceeded the calculated minimum sample size, thereby increasing the statistical power.

The demographic, medical, and dental information collected included: gender, age, education level, occupation, presence of systemic disease, frequency of tooth brushing and interdental cleaning, smoking and alcohol habits, history of periodontal treatment prior to implant surgery, plaque control education, frequency of supportive care, history of bruxism, implant brand, and time elapsed after functional loading.

### Clinical evaluation

The clinical evaluation form recorded the type of prosthetic retention (screw-retained or cement-retained), prosthesis fit, increased cement presence, number and location of implants, mechanical complications, VD, KM width and phenotype, presence of suppuration, history of periodontitis, and periodontal parameters (mPI, mGI, PD, and BOP). Peri-implant soft tissue parameters were recorded using the UNC-15 periodontal probe (PCPUNC15; Hu-Friedy, Chicago, IL, USA).

All clinical measurements (including VD and KM width) were performed by a single evaluator following a standardized protocol. To reduce measurement variability, each measurement was repeated 2–3 times during the same session, and the average value was recorded. When the difference between measurements was significant, additional measurements were taken and the most consistent measurements were used. This approach aimed to increase the repeatability of measurements by reducing random error caused by the evaluator.

**Fig. 1:**
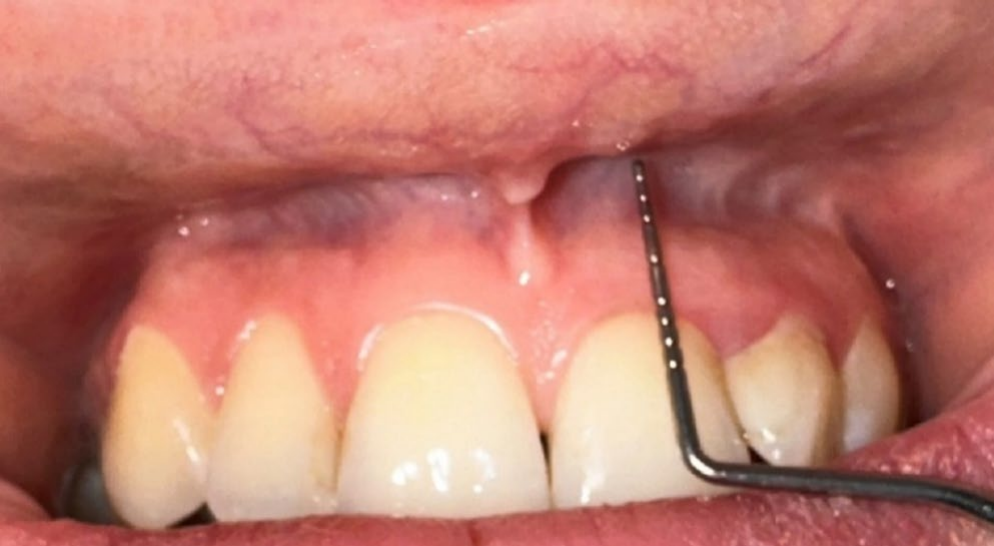
Measurement of vestibular depth
- **Supuration**: Assessed by advancing the periodontal probe into the peri-implant sulcus; presence recorded as “+”, absence as “–” [27].

- **mGI**: Peri-implant soft tissue inflammation was assessed using mGI as defined by Lobene et al. [18]. The applicability of mGI in peri-implant soft tissue monitoring has also been noted in recent evidence syntheses [19].
- **mPI**: Plaque accumulation was assessed using mPI as proposed by Mombelli et al. [19, 20].
- **PD**: Total mouth PD was measured by positioning the periodontal probe parallel to the long axis of the implant and applying only the weight of the probe itself. The coronal margin of the gingiva was considered the coronal limit, and the pocket base was considered the apical limit. Measurements were taken at six points (mesiobuccal, buccal, distobuccal, mesiolingual/palatal, lingual/palatal, distolingual/palatal) for each implant, and the average values per implant were recorded [21].
- **BOP**: Periodontal probing was performed circumferentially around the implant using a probing force of approximately 0.20–0.25 N. Its presence was recorded dichotomously (yes/no; 1/0) and standardized using the 4-surface (mesial, distal, buccal, lingual) protocol commonly used in clinical practice. BOP percentages were calculated on an implant basis [3].
- **KM width**: KM width was measured with a periodontal probe from the gingival margin to the mucogingival junction at the mesiobuccal, midbuccal, and distobuccal points on the buccal surface of each implant; the mean of the three measurements was used in the analyses, and KM width was categorized as < 2 mm or ≥ 2 mm [11, 22].
- **KM thickness**: This was determined by placing the periodontal probe into the sulcus and assessing the visibility of the probe tip; it was classified as thin (< 2 mm) or thick (≥ 2 mm) [23, 24].
- **VD**: Defined as the distance between the coronal border of the attached gingiva and the deepest point of the mucobuccal fold (Fig. 1) [13]. Measurements were taken at the mid-buccal point of each implant; the lip/cheek was gently retracted to reveal the mucobuccal fold, taking care not to artificially increase the anatomical depth by stretching the tissue [13]. VD was recorded in millimeters and evaluated as both a continuous variable and a categorical variable in the analyses. In categorical classification, implants were grouped as shallow VD (≤ 4 mm) and adequate VD (> 4 mm) [13, 25, 26]. The four-millimeter threshold value was determined by considering the basic approaches used in the literature [13, 25] and the fact that the finding retained its significance in threshold sensitivity analyses conducted in the 2–6 mm range.

### Diagnostic criteria

 The diagnoses of peri-implant health, peri-implant mucositis, and peri-implantitis were determined based on the criteria proposed in the 2017 World Classification of Periodontal and Peri-implant Diseases.

*Peri-implant health* was defined as the absence of clinical signs of inflammation in the peri-implant mucosa (no erythema, no edema), no BOP and no suppuration, no pathological pocket depth (PD ≤ 5 mm), and a MBL < 3 mm relative to the implant shoulder [5].

*Peri-implant mucositis* was defined as the presence of clinical signs of inflammation (erythema, edema) in the peri-implant mucosa and positive BOP, combined with MBL suggestive of bone loss on radiographs that remained below the peri-implantitis threshold. Due to limited baseline radiographs, implants with MBL < 3 mm relative to the implant shoulder but with BOP were classified as peri-implant mucositis [5].

*Peri-implantitis* is defined by the presence of clinical signs of inflammation in the peri-implant mucosa (BOP and/or suppuration), along with increased pocket depth (PD ≥ 6 mm) and radiographic evidence of MBL ≥ 3 mm apical to the implant shoulder. In cases where previous radiographs were not systematically available, in line with the 2017 World Workshop recommendations, PD ≥ 6 mm and MBL ≥ 3 mm from the implant shoulder, together with BOP and/or suppuration, were considered as peri-implantitis [5].

###  Radiographic evaluation

 Panoramic radiographs taken within 3 months prior to clinical examination (Planmeca Promax 2D, Planmeca, Helsinki, Finland; 68 kVp, 16 mA, 13 s) were evaluated. MBL was measured in the mesial and distal regions using the distance from the implant shoulder to the MBL, and measurements were performed using ImageJ software (Rasband, W.S., ImageJ, U.S. National Institutes of Health, Bethesda, Maryland, USA). The mean of the mesial and distal values was calculated per implant [28]. To increase the accuracy of the measurements and reduce possible scale differences in the panoramic images, calibration (scaling) was performed in ImageJ (ImageJ v1.53, NIH, USA) using the known length of an implant located in the mouth as a reference. All measurements were performed by a single calibrated researcher using a standard protocol.

###  Statistical analysis

The unit of analysis is the implant; observations are clustered within patients due to the presence of multiple implants in the same patient. The distribution of continuous variables was assessed using the Shapiro–Wilk test and visual methods (histogram/Q–Q). Variables following a normal distribution were presented as mean ± SD, those not following a normal distribution as median (min–max), and categorical variables as n (%). Binary comparisons between VD groups were performed exploratively using the Mann–Whitney U and Pearson χ² tests; main inferences were reported using GEE logistic regression models that accounted for within-patient correlation. For peri-implantitis (yes/no), GEE was set up with a binomial distribution–logit link; the working correlation was selected as exchangeable, and robust (sandwich) standard errors were used (cluster: patient). Clinically meaningful/a priori determined variables were included in the model; multicollinearity in the model was controlled with VIF/tolerance. A Type I error of 5% (two-tailed) was accepted. SPSS (IBM Corp. Released 2017. IBM SPSS Statistics for Windows, Version 25.0. Armonk, NY: IBM Corp.) software was used for statistical analyses.

###  Findings

The mean age of the 65 patients included in the study was 48.18 ± 10.36 years, and the median age was determined to be 50 (19–68). Fifty-eight point 5% of the participants were female, and 41.5% were male. Thirty point 8% of the patients smoked, and 69.2% did not smoke. A history of periodontitis was determined in 38.5% of the patients, and a history of periodontal treatment was determined in 49.2% of the patients. Oral hygiene levels were low in 30.8%, moderate in 27.7%, and good in 41.5%. A total of 336 implants were evaluated in 65 patients; the number of implants per patient ranged from 1 to 15, with an average of 5.17 ± 3.15 and a median of 5.

When implant sites were examined, 9.5% of implants were placed in the maxillary anterior region, 36.3% in the maxillary posterior region, 7.4% in the mandibular anterior region, and 46.7% in the mandibular posterior region. The duration of implant function was 1–5 years in 48.5%, 5–10 years in 39.9%, and > 10 years in 11.6%. The mean mPI was 1.21 ± 0.63, mGI 1.11 ± 0.66, BOP 45.68 ± 42.49, and PD 3.78 ± 1.59 mm. Crest width was < 2 mm in 52.7% of implants and ≥ 2 mm in 47.3%; crest thickness was determined to be thin in 31% and thick in 69%. Supuration was observed in 19.6%. The mean gingival recession was 0.04 ± 0.21 mm, and the radiographic bone loss was calculated as 0.87 ± 1.58 mm (median: 0; min–max: 0–7.5). When evaluated according to clinical diagnostic criteria, peri-implant health was found in 39.9% (*n* = 134) of implants, peri-implant mucositis in 34.5% (*n* = 116), and peri-implantitis in 25.6% (*n* = 86). In terms of VD, VD ≤ 4 mm was found in 26.5% of implants, and VD > 4 mm was found in 73.5% (Table 1). When evaluated at the patient level, 1 of 65 patients (1.5%) had only implants with VD ≤ 4 mm, 31 (47.7%) had only implants with VD > 4 mm, while 33 patients (50.8%) had a “mixed” distribution of both VD ≤ 4 mm and VD > 4 mm implants within the same individual.

**Table 1:** Demographic characteristics of patients and general data on implants

	Mean	Sd	Median	Min.	Max.
Age (years)	48.18	10.36	50	19	68
Gender					
• Total n (%)	65 (100%)				
• Female n (%)	38 (58.5%)				
• Male n (%)	27 (41.5%)				
Smoking n (%)					
• Non-smoker n (%)	45 (69.2%)				
• Smoker n (%)	20 (30.8%)				
History of Periodontitis n (%)	25 (38.5%)				
History of Periodontal Treatment n (%)	32 (49.2%)				
Oral Hygiene n (%)					
• Poor n (%)	20 (30.8%)				
• Moderate n (%)	18 (27.7%)				
• Good n (%)	27 (41.5%)				
Implant Site n (%)					
• Anterior Maxilla n (%)	32 (9.5%)				
• Posterior Maxilla n (%)	122 (36.3%)				
• Anterior Mandible n (%)	25 (7.4%)				
• Posterior Mandible n (%)	157 (46.7%)				
Implant Duration n (%)					
• 1–5 years n (%)	163 (48.5%)				
• 5–10 years n (%)	134 (39.9%)				
• > 10 years n (%)	39 (11.6%)				
mPI	1.21	0.63	1.25	0	3
Mgi	1.11	0.66	1.25	0	2
BOP (%)	45.68	42.49	50	0	100
PD	3.78	1.59	3.3	1.6	10.5
KM Width n (%)					
• < 2 n (%)	177 (52.7%)				
• ≥ 2 n (%)	159 (47.3%)				
KM Thickness n (%)					
• Thin n (%)	104 (31%)				
• Thick n (%)	232 (69%)				
Suppuration n (%)	66 (19.6%)				
Gingival Recession mm	0.04	0.21	0	0	1.5
Radiographic Bone Loss mm	0.87	1.58	0	0	7.5
Diagnosis n (%)					
• Peri-implant health n (%)	134 (39.9%)				
• Peri-implant mucositis n (%)	116 (34.5%)				
• Peri-implantitis n (%)	86 (25.6%)				
VD n (%)					
• ≤ 4	89 (26.5%)				
• > 4	247 (73.5%)				
Number of implants per patient	5,17	3,15	5	1	15

Data are expressed as mean ± standard deviation, median (minimum: maximum), and n (%)

 In implants with shallow VD, mPI, mGI, BOP, and PD values were found to be higher in exploratory bivariate analyses compared to those with adequate VD (all p<0.05; Table 2).

**Table 2:** Comparison of clinical and radiographic parameters according to VD groups

	VD		*p* value
	≤ 4	> 4	
mPI	1.44 ± 0.54 1.25(0.25–3.25)	1.13 ± 0.64 1(0–3)	**< 0.001**
mGI	1.26 ± 0.59 1.25(0–2)	1.05 ± 0.68 1.25(0–2)	**0.014**
BOP (%)	53.93 ± 41.27 50(0–100)	42.71 ± 42.62 50(0–100)	**0.023**
PD	3.99 ± 1.45 3.6(2.25–8.25)	3.7 ± 1.63 3.3(1.6–10.5)	**0.041**
Smoking n (%)			
• Non-smoker n (%)	48(53.9%)	168(68%)	**0.017**
• Smoker n (%)	41(46.1%)	79(32%)	
History of Periodontitis n (%)			
• Present n (%)	59(66.3%)	144(58.3%)	0.186
• Absent n (%)	30(33.7%)	103(41.7%)	
KM Width n (%)			
• <2 n (%)	77(86.5%)	100(40.5%)	**< 0.001**
• ≥2 n (%)	12(13.5%)	147(59.5%)	
KM Thickness n (%)			
• Thin n (%)	52(58.4%)	52(21.1%)	**< 0.001**
• Thick n (%)	37(41.6%)	195(78.9%)	
Gingival Recession	0.09 ± 0.29 0(0–1.5.5)	0.03 ± 0.17 0(0–1.5.5)	**< 0.001**
Radiographic bone loss	1.05 ± 1.39 0(0–5)	0.79 ± 1.64 0(0–7.5.5)	**0.010**
Suppuration n (%)	29(32.6%)	37(15%)	**< 0.001**
Diagnosis n (%)			
• Peri-implant health n (%)	25(28.1%)	109(44.1%)	**0.003**
• Peri-implant mucositis n (%)	30(33.7%)	86(34.8%)	
• Peri-implantitis n (%)	34(38.2%)	52(21.1%)	

Data are expressed as mean ± standard deviation, median (minimum: maximum), and n (%)

For continuous variables: Mann–Whitney U test; for categorical variables: Pearson χ² test was used

The p-values given in this table are exploratory comparisons at the implant level; within-patient clustering was not taken into account

 When evaluated in terms of smoking, the proportion of smokers was higher in the shallow VD group (p=0.017). No significant difference was found between the VD groups in terms of periodontitis history (p=0.186) (Table 2).

 The proportion of implants with KM width <2 mm was significantly higher in the shallow VD group, while ≥2 mm KM and thick mucosa phenotype were observed at a higher rate in the adequate VD group (p<0.001 for both; Table 2).

 In the shallow VD group, gingival recession and radiographic MBL values, as well as the prevalence of suppuration, were found to be higher in exploratory bivariate analyses compared to the adequate VD group (all p<0.05; Table 2).

 When the distribution of diagnoses was examined according to peri-implant disease status (health, peri-implant mucositis, peri-implantitis), it was determined that peri-implant health was more prevalent in the sufficient VD group, while peri-implantitis was more common in the shallow VD group (p=0.003) (Table 2).

 The relationship between VD and peri-implantitis was tested in two ways: (i) categorical threshold model (≤4 mm vs >4 mm; Table 3) and (ii) continuous model (mm; Table 4).

**Table 3:** GEE multivariate logistic regression model for peri-implantitis determinants

				**95% confidence interval for OR**	
	**B**	**OR**	**p value**	**Lower**	**Upper**
Age	0.015	1.016	0.469	0.974	1.059
VD (≤4 mm) *	0.877	**2.405**	**0.010**	1.233	4.690
Smoking (current) *	0.735	2.086	0.137	0.791	5.495
History of periodontitis (present) *	0.536	1.710	0.247	0.690	4.235
Implant duration (1-5 years) *					
• 5-10 years	1.393	**4.026**	**0.044**	1.037	15.622
• >10 years	1.084	2.956	0.113	0.775	11.274
Implant site (Mandibular posterior) *					
• Maxilla anterior	-0.894	**0.409**	**0.026**	0.186	0.901
• Maxilla posterior	-0.474	0.623	0.059	0.381	1.017
• Mandible anterior	-0.454	0.635	0.354	0.243	1.659
Oral hygiene (poor)*					
• Moderate	-0.621	0.537	0.019	0.315	0.915
• Good	-1.876	0.153	<0.001	0.054	0.432
KM width (<2 mm) *	-0.310	0.734	0.479	0.311	1.728
KM thickness (Thin)*	-0.525	0.592	0.245	0.245	1.432

OR = Exp(B). GEE analysis was performed using the binomial distribution–logit link, the working correlation was selected as exchangeable, and robust (sandwich) standard errors were used with implants clustered within patients

The reference categories in the GEE model were, respectively: VD > 4 mm, smoking status (non-smoker), history of periodontitis (none), implant duration 1–5 years, implant site mandibular posterior, poor oral hygiene, KM width ≥ 2 mm, and thick KM thickness

*: OR for categorical variables was calculated relative to the reference categories in the table

**Table 4:** Relationship between VD and peri-implantitis as continuous variables: multivariate GEE model

Variable	B (Coefficient)		OR (Odds Ratio)	*p* value	95% Confidence Interval (OR)
VD (mm)		−0.124	**0.883**	**0.034**	0.788–0.990
Smoking (Current)*		−0.183	0.833	0.736	0.287–2.421
Age		−0.037	0.964	0.230	0.908–1.023
KM Width (< 2 mm)*		0.155	1.167	0.776	0.403–3.380
KM Thickness (Thin)*		0.726	2.067	0.144	0.780–5.479
History of Periodontitis (Present)*		0.688	1.99	0.192	0.708–5.596
Implant Duration*					
• 5–10 years		0.676	1.966	0.251	0.619–6.246
• >10 years		1.949	**7.019**	**0.005**	1.775–27.743
Implant Site*					
• Maxillary Anterior		−0.283	0.753	0.755	0.127–4.464
• Maxillary Posterior		−1.104	**0.332**	**0.005**	0.155–0.711
• Mandibular Anterior		−0.614	0.541	0.175	0.223–1.314
Oral Hygiene*					
• Poor		2.023	**7.561**	**0.001**	2.241–25.508
• Moderate		0.363	1.438	0.591	0.382–5.409

*Reference Categories: Smoking (Non-smoker), Gingival Margin Width (≥ 2 mm), Gingival Margin Thickness (Thick), History of Periodontitis (None), Implant Duration (1–5 years), Implant Site (Posterior Mandible), Oral Hygiene (Good). OR > 1 indicates higher odds, OR < 1 indicates lower odds; *p* < 0.05 significant

 In the GEE multivariate logistic regression model, VD was found to be independently associated with peri-implantitis. When the reference category was set as VD >4 mm, the odds of peri-implantitis were significantly higher in implants with VD ≤4 mm (OR=2.405; 95% CI: 1.233–4.690; p=0.010) (Table 3).

 Although the implant time variable was not significant overall (p=0.132), the 5–10 year category was found to increase the likelihood of peri-implantitis compared to the reference category (OR=4.026; 95% CI: 1.037–15.622; p=0.044). The implant region variable showed borderline significance at the overall level (p=0.084); in subgroup comparisons, the maxilla anterior region was associated with a lower probability of peri-implantitis compared to the reference region (OR=0.409; 95% CI: 0.186–0.901; p=0.026). A statistically significant relationship was found between oral hygiene level and peri-implantitis (p=0.001). With the reference category being “poor” oral hygiene, the probability of disease was significantly lower in the group with good hygiene (OR=0.153, p<0.001), and the risk was also reduced in those with moderate hygiene compared to the reference group (OR=0.537, p=0.019) (Table 3).

 Smoking (p=0.137), history of periodontitis (p=0.247), mucosal width (p=0.479), mucosal thickness (p=0.245), and age (p=0.469) were not statistically significant in the multivariate model (Table 3).

 According to the results of the multivariate GEE analysis, VD, when evaluated in mm, was determined to be an independent factor that significantly affected the odds of peri-implantitis (p=0.034). Even after including and controlling for clinical confounders such as age, KM characteristics, implant function duration, and oral hygiene in the model, the protective effect of VD remained statistically significant (Table 4).

 The OR (0.883) obtained from the model indicates that each 1-mm increase in VD is associated with an approximately 11.7% decrease in the odds of peri-implantitis. This finding indicates that VD provides a gradual protection on peri-implant health in inverse proportion to the depth amount, not only at a fixed threshold value (e.g., 4 mm) (Table 4).

 The analysis also revealed that implants with a functional lifespan of more than 10 years (OR=7.019, p=0.005) and low oral hygiene levels (OR=7.561, p=0.001) were the factors that most increased the odds of peri-implantitis. These results suggest that VD may represent an important anatomical component within the cumulative set of factors linked to peri-implant disease (Table 4).

To evaluate the robustness of the 4 mm cut-off point used for VD, threshold sensitivity (robustness) analysis was performed by establishing separate GEE logistic regression models with alternative threshold values in the range of 2–6 mm (Table 5). In these analyses, the VD ≤4 mm threshold showed a significant association with peri-implantitis (OR=2.32; p=0.025) and provided a practical classification for clinical application. Although the VD ≤2 mm threshold was also found to be significant (OR=2.72; p=0.023), the 4 mm threshold was preferred in terms of classification balance and clinical applicability due to its narrower subgroup definition.

**Table 5:** Threshold sensitivity analysis for VD cut-off points

Tested Threshold Value	OR	*p* Value	Decision
VD ≤ 2 mm	2,72	0,023	-
VD ≤ 3 mm	2,04	0,083	-
VD ≤ 4 mm	**2**,**32**	**0**,**025**	**Optimal cut-off**
VD ≤ 5 mm	1,92	0,074	-
VD ≤ 6 mm	1,73	0,111	-

*Analyses were performed using separate GEE logistic regression models for each threshold value

##  Discussion

 In our study, the prevalence of peri-implantitis was found to be 25.6% (n=86) and the rate of shallow VD was 26.5% (n=89), highlighting the clinical significance of the problem. These findings indicate that in clinical practice, peri-implantitis is encountered in approximately one in every four implants, and similarly, shallow VD is encountered in approximately one in every four implants. Furthermore, the prevalence of peri-implantitis in the shallow VD group was 38.2%, and in the multivariate GEE analysis (categorical threshold model: VD≤4 mm), shallow VD showed an independent association with peri-implantitis (OR=2.405; 95% CI: 1.233–4.690; p=0.010) supports the notion that VD may be a clinically important anatomical component in maintaining peri-implant health.

 The OR of 2.40 identified for shallow VD can be clinically interpreted as follows: the odds of peri-implantitis occurring in implants with shallow VD are approximately 2.4 times higher than in implants with adequate VD. This finding suggests that a shallow vestibule is not merely a condition that complicates oral hygiene practices; it may be independently associated with peri-implantitis even when other factors are controlled for in a multivariate model.

 The presence of KM is generally considered advantageous for peri-implant tissues; although evidence is heterogeneous, increasing data suggest that a width of ≥2 mm of KM around implants may be associated with lower plaque indices, reduced inflammation, and minimal marginal bone loss [28–32]. In this study, exploratory bivariate analyses showed that implants with shallow VD had a significantly higher proportion of KM width <2 mm (p<0.001), while adequate VD was associated with a higher prevalence of thick mucosa (p<0.001). Halperin-Sternfeld et al. [13], reported in their 6-year longitudinal follow-up studies that shallow VD regions had a gingival margin band approximately 1 mm narrower than regions with adequate VD. Similarly, Monje and Blasi [33], showed that 95.7% of implant regions with gingival margin width <2 mm had shallow VD. Current systematic reviews and clinical studies have shown that a KM width <2 mm, especially when combined with a thin gingival phenotype, may be associated with gingival recession, MBL, plaque accumulation, and an increased risk of peri-implantitis. Furthermore, a narrow KM has been associated with greater discomfort during brushing [34–38]. Other studies have demonstrated that pro-inflammatory cytokine levels are significantly higher in peri-implant regions with a thin soft tissue phenotype [39, 40]. Reduced stability of peri-implant soft tissues in areas with both shallow VD and insufficient KM may promote plaque retention and bacterial colonization, thereby increasing the microbial load in peri-implant pockets; this is consistent with the findings of our study.

 Although VD, CM width, and mucosal thickness are biologically related, our multivariate GEE analysis identified VD≤4 mm (categorical threshold model) as the only soft tissue parameter independently and significantly associated with peri-implantitis. This finding suggests that shallow VD may not merely be a reflection of other soft tissue characteristics and may be a clinically unique risk indicator. Although gingival margin width was not found to be an independent risk indicator in the model, shallow VD cases are often seen in conjunction with insufficient gingival margin width in clinical practice; therefore, a comprehensive soft tissue approach should be adopted in evaluation and treatment planning.

 In our study, implants with shallow VD had higher mean mPI, mGI, BOP, and PD values compared to implants with adequate VD in exploratory bivariate analyses (p<0.001, p=0.014, p=0.022, and p=0.032, respectively). Halperin-Sternfeld et al. [13], reported that shallow VD was strongly associated with a narrow gingival margin, poor oral hygiene, and adverse peri-implant health outcomes at 6-year follow-up. Similarly, an increase in VD has been reported to be associated with lower mPI and mGI scores [41]. Furthermore, Di Leone et al. [42] also reported that VD was significantly associated with accumulation (mPI) at the implant level. It is known that shallow vestibules facilitate plaque accumulation, predisposing to gingival inflammation, gingival recession, and discomfort during oral hygiene. Previous reports have shown that shallow VD impedes plaque control and is closely related to inflammation, findings that are consistent with our study.

 In our study, the amount of gingival recession in the shallow VD group was found to be statistically significantly greater than in the adequate VD group. A previous study focused on the relationship between inadequate VD and increased gingival recession in natural teeth [14]. Shallow vestibules have been reported to predispose to gingival recession [43]. Kadkhodazadeh et al. [44], emphasized that maximum soft tissue recession occurred in areas where thin biotype and shallow VD were present together. Insufficient VD may contribute to recession directly through muscle traction, while also indirectly increasing recession through the difficulty of oral hygiene procedures and soft tissue trauma. Indeed, Iorio-Siciliano et al. [45], reported that adequate height of keratinized tissues is critical for the long-term stability of peri-implant marginal tissues; they emphasized that adequate KM height preserves tissue health by facilitating biofilm control and reducing discomfort during brushing. These data support, from a biomechanical and hygienic perspective, why the shallow VD and accompanying narrow KM band detected in our study are associated with a higher rate of gingival recession and inflammation.

 The mean radiographic bone loss was also found to be significantly higher in the shallow VD group. Consistent with our study, Halperin-Sternfeld et al. [13], reported greater radiographic bone loss in shallow VD areas. Some studies have suggested that soft tissue grafting around the implant may reduce MBL, biofilm accumulation, and peri-implant inflammation [39, 46]. Therefore, in areas with shallow VD and soft tissue insufficiency, procedures such as vestibuloplasty or soft tissue grafting may be considered in selected cases to help prevent peri-implant bone loss.

 This study demonstrated that peri-implant health was significantly higher in areas with adequate VD, while peri-implantitis was more common in areas with shallow VD. It has been suggested that a shallow vestibule may increase the risk of peri-implantitis by limiting toothbrush access to the mucosal margin of implant-supported restorations [47]. A recent cross-sectional study also reported an association between shallow VD (≤4 mm), absence of attached mucosa, and peri-implantitis [48]. Kadkhodazadeh et al. [44], demonstrated a significant association between plaque accumulation, shallow VD, and peri-implantitis in their retrospective study involving 139 implants. In our multivariate GEE analysis, VD≤4 mm was found to be independently associated with peri-implantitis even when age, smoking, KM width, and mucosal thickness were controlled for. Considering all these findings together, it can be said that shallow VD may be a clinical peri-implant phenotype component associated with peri-implantitis.

 One of the most striking findings of our study is the linear relationship between VD and the odds of peri-implantitis. VD was found to be statistically significant not only as a categorical threshold value (4 mm and below) but also when analyzed in a multivariate GEE (continuous model: VD, mm) (p = 0.034). According to our multivariate analysis results, a 1 mm increase in VD was associated with an approximately 11.7% decrease in the odds of peri-implantitis (OR = 0.883). This finding supports that the effect of VD is not limited to a fixed “breakpoint” but is associated with a protective mechanism directly related to the amount of depth.

 There are significant anatomical differences in VD between the maxillary anterior and mandibular posterior regions; our findings indicate that VD is generally wider in the maxillary anterior, whereas in the mandibular posterior it is frequently ≤4 mm due to anatomical/muscular connections. However, despite the inclusion of the implant site in the GEE model, the persistence of an independent association between shallow VD and peri-implantitis suggests that this relationship may not be explained solely by regional anatomy and that shallow VD may be a clinically significant risk indicator. Therefore, careful evaluation of soft tissue conditions in implant planning, particularly in regions such as the posterior mandible, and consideration of approaches to increase VD in selected cases may be appropriate.

 Insufficient VD, when combined with insufficient KM width, can further compromise oral hygiene by increasing food impaction during mastication. Consequently, correction of shallow vestibules may be considered to facilitate effective oral hygiene and ensure long-term peri-implant health [49]. While the current literature mostly focuses on KM width and the general peri-implant phenotype, this study considers VD as a specific component of the peri-implant soft tissue phenotype and demonstrates that it is a risk indicator that shows an independent relationship with peri-implantitis even when other soft tissue parameters are controlled.

 This study has some limitations. The retrospective and cross-sectional design prevents establishing a causal relationship between VD and peri-implantitis. Since peri-implant soft tissue health and implant success are influenced by numerous local and systemic factors, it is difficult to completely isolate the independent contribution of VD. Although calibrated with ImageJ, the use of panoramic radiographs in MBL assessment may limit measurement accuracy compared to periapical or 3D imaging. The small sample size in the shallow VD and KM=0 mm groups necessitated categorization (e.g., KM <2 mm), which may affect generalizability. Although intra-observer reliability (ICC/Kappa) was not formally analyzed, all measurements were performed by a single experienced expert to minimize variability. Finally, excluding restorations that obstruct PD measurement may lead to selection bias and limited generalizability. However, considering that observations in patients with multiple implants are not independent, the use of the GEE approach can be considered an important aspect that increases the power of statistical modeling.

##  Conclusion

 The findings of this study suggest that shallow VD may be associated with peri-implant health indicators and the presence of peri-implantitis. Implants in areas with shallow VD showed significantly more plaque accumulation, gingival inflammation, bleeding, and radiographic bone loss compared to areas with adequate VD. Furthermore, shallow VD may be associated with mucosal trauma and gingival recession, along with difficulties in maintaining adequate oral hygiene. Overall, the combination of shallow VD and inadequate KM appears to be associated with a phenotype in which peri-implantitis is more frequently observed. However, larger-scale, prospective, and longitudinal studies are needed to confirm the causality and clinical implications of these observed associations.

## Data Availability

The datasets used and/or analyzed in the present study are not publicly available due to patient confidentiality; however, they can be obtained from the corresponding author upon reasonable request.

## Abbreviations

VD: Vestibular Depth
KM: Keratinized Mucosa
PD: Probing Depth
BOP: Bleeding on Probing
mPI: Modified Plaque Index
mGI: Modified Gingival Index
GEE: Generalized Estimating Equations
MBL: Marginal Bone Loss
OR: Odds Ratio
